# Care transition and network activation in Portugal

**Published:** 2012-09-04

**Authors:** Silvina Santana, M Viana

**Affiliations:** Associate Professor with Agregação, Department of Economics, Management and Industrial Engineering, Institute of Electronics Engineering and Telematics of Aveiro, and Research Unit in Governance, Competitiveness and Public Policies, University of Aveiro, Portugal; Institute of Electronics Engineering and Telematics of Aveiro, Department of Economics, Management and Industrial Engineering, University of Aveiro, Aveiro, Portugal

**Keywords:** care transition quality, care network activation, patient perceptions, stroke rehabilitation, health and social care services, homecare, RCT, Portugal

## Abstract

**Purpose:**

To report on the use of a user-centred model and methodology to assess the quality of care transition and network activation action, in light of an ongoing home supported discharge procedure for stroke patients in Portugal.

**Theory:**

In Portugal, the health care system presents weaknesses resulting from a remarkable diversity of entry points, inadequate use of scarce and expensive resources and difficult information flow between institutions and professionals. The social care network is mostly run by privately owned, non-profit-making institutions (IPSS and Misericórdias), that operate close to the populations. In 2006, the Portuguese government has created the National Network of Continuous Integrated Care (RNCCI) that has been presented as a third level of care, connecting with acute care hospitals and health centres. The network is based on establishing protocols with existing institutions (many of them IPSS or hospitals belonging to Misericórdias). Home care is supposed to be one important element in this network, but implementation only now has started. Integration outside the RNCCI (e.g. between hospitals and primary care) and between the RNCCI and other levels of care is still weak. In this context, researching and evaluating the interfaces among the entities that populate the networks is of fundamental importance. Surprisingly, this is one of the less focused aspects in integrated care research. Some indicators have been used, but they are manifestly insufficient. Above all, they represent disentangled attempts to monitor individual services responsiveness in the absence of a robust methodology, designed to provide a more holistic perspective of performance and self-improving capabilities. In this study we present the preliminary results of a study designed to assess the quality of care transition and network activation action, from the patient’s perspective.

**Methods:**

We have studied the whole patient’s course, from the admission to the stroke unit to six months after discharge. A methodology has been developed to assess the quality of care transitions and network activation actions.

**Results and conclusions:**

Four fundamental aspects have been researched at the moment of patient discharge from the several units involved in health care: dealing with medication at home, dealing with activities of daily living, finding help in community and dealing with the moment of discharge itself. More precisely, we have addressed patient self-reported ability to deal with these situations, the level of information provided to the patient by the heath unit at discharge and the way the process was conducted. Additionally, we have investigated the way each node in the network provides the patient with an anchor contact point in the discharging unit, a referring contact point in the destination unit, a written care summary and plan and a discharge letter addressed to the patient’s family doctor. Finally, we addressed the role of the family doctor as gate kipper.

Powerpoint presentation available at http://www.integratedcare.org at congresses – San Marino – programme.

